# Spectrum of Endoscopic Findings in Patients of Upper Gastrointestinal Bleeding at a Tertiary Care Hospital

**DOI:** 10.7759/cureus.4562

**Published:** 2019-04-29

**Authors:** Waseem Sarwar Malghani, Romaisa Malik, Farooq Mohyud Din Chaudhary, Asim Tameez Ud Din, Misbah Shahid, Sultan Ahmad, Asma Tameez Ud Din, Sana Mohyud Din Chaudhary

**Affiliations:** 1 Gastroenterology, Nishtar Medical University & Hospital, Multan, PAK; 2 Internal Medicine, Rawalpindi Medical University, Rawalpindi, PAK; 3 Internal Medicine, Nishtar Medical University & Hospital, Multan, PAK; 4 Internal Medicine, Combined Military Hospital Lahore Medical College and Institute of Dentistry, Lahore, PAK

**Keywords:** esophageal varices, upper gastrointestinal bleeding, esophageal varices, esophagogastroduodenoscopy, endoscopic findings, peptic ulcer

## Abstract

Background & aims

Upper gastrointestinal bleeding (UGIB) is a common medical emergency that results in high patient morbidity and mortality. There are numerous causes of UGIB. The aim of our study was to evaluate the endoscopic findings in patients of UGIB in this part of the world.

Methods

This retrospective study was conducted at the Department of Gastroenterology Nishtar Medical University & Hospital Multan from June 2018 to March 2019. Record of all patients undergoing esophagogastroduodenoscopy (EGD) for evaluation of UGIB was reviewed. Data was entered and analyzed using SPSS version 20 (IBM, Armonk, NY, USA).

Results

Record of 730 (464 male and 266 females) patients undergoing EGD for UGIB was reviewed. Mean age of study population was 49.38 years with standard deviation of 14.86 years. Age of the youngest patient was 14 years while the oldest patient was 99 years of age. More than half of the patients (53%) belonged to the 41-60 years age group. The most common endoscopic finding was esophageal varices in 371 (50.8%) patients, followed by gastropathy (114, 15.6%), gastritis (68, 9.3%), cardio-fundal varices (58, 7.9%) and duodenal ulcer (26, 3.6%). Esophageal varices, gastritis, duodenal ulcers and gastric carcinomas were more likely to be found in male UGIB patients as compared to female patients (p = 0.039). Gastropathy, esophageal ulcer and gastric ulcer were more likely to be found in female UGIB patients. Esophageal varices and cardio-fundal varices were more likely to be found in UGIB patients from middle age group (p = 0.000). Whereas gastritis, duodenal ulcer, gastric erosions and duodenitis were more likely to be found in older (>60 years) UGIB patients (p = 0.000).

Conclusion

UGIB was more likely to occur in male gender. In our study, bleeding from esophageal varices was the most important cause of UGIB in this part of the world and bleeding from duodenal ulcer was quite uncommon as compared to the western world. Variceal bleeding had a significant association with male gender and middle age group patients. While duodenal ulcer bleed had a significant association with older age.

## Introduction

Upper gastrointestinal bleeding (UGIB) is a very common and serious medical problem encountered in the Accident and Emergency Department of hospitals around the world. This condition has a high mortality of around 5-15% [1,2] and therefore requires immediate resuscitation and intervention. It is more common in males as compared to females. However, the mortality rate is equal in both genders [3]. UGIB occurs when the bleeding site is proximal to ligament of Treitz. Hematemesis or blood in vomitus is the presenting complaint in 40-50% cases of UGIB [4]. In approximately 80% of patients bleeding stops spontaneously without any intervention. In the rest of patients who continue to bleed, they have a high rate of mortality and require intervention to stop the bleeding.

There is a long list of causes that result in upper gastrointestinal bleeding. Prominent causes include peptic ulcer disease (PUD), bleeding esophageal varices, esophagitis and gastric and duodenal erosions. There are some less common causes of UGIB as well. These include Dieulafoy's lesions, angiodysplasia, aorto-enteric fistula and hemobilia. Bleeding from esophageal varices is a very important cause of UGIB which has a high morbidity and mortality. Esophageal varices are found in lower one-third of esophagus as abnormally dilated submucosal veins. These usually occur as a result of cirrhosis-related complication of portal hypertension [5]. In this region of the world, bleeding from the esophageal varices is the commonest cause of UGIB [6]. This contrasts to the western world where peptic ulcer disease is more common.

Esophagogastroduodenoscopy (EGD) is an indispensable tool in establishing the cause and site of bleeding in patients of UGIB. It also has the advantage of permitting therapeutic interventions like endoscopic variceal band ligation for bleeding varices, clipping and adrenaline injection for bleeding peptic ulcers and biopsies of any suspicious looking area. The purpose of our study was to ascertain the different causes of UGIB in this part of the world.

## Materials and methods

This retrospective study was carried out at the Department of Gastroenterology Nishtar Medical University & Hospital Multan after approval from the Institutional Ethical Review Committee of Nishtar Medical University Multan. This study included all patients referred to endoscopy room from both the hospital's own wards as well as the surrounding hospitals and clinics from June 2018 to March 2019. UGIB was labeled if a patient had complaints of hematemesis or of passing tarry black stools (melena). EGD was performed in 730 patients after resuscitation and consent by a consultant gastroenterologist having at least three years post fellowship experience. EGD was performed in the endoscopic room under lignocaine gargles.

Data regarding demographics (age, gender) and endoscopic findings were collected. Data were then entered into SPSS version 20 (IBN, Armonk, NY, USA). Statistical data analysis was performed with chi-square. Statistical significance was determined at p < 0.05. Information obtained was then analyzed according to age, gender and endoscopic findings and presented in form of tables.

## Results

Our study was comprised of 730 patients who presented with UGIB, of which 464 (63.6%) were males and 266 (36.4%) were females. Male to female ratio was 1.7:1. Mean age of study population was 49.38 years with standard deviation of 14.86 years. Mean age of males was 49 years while that of females was 50 years. Age of the youngest patient was 14 years while the oldest patient was 99 years of age. Maximum number of patients (n = 203) belonged to the fifth decade of life. More than half of the patients (53%) who had UGIB belonged to the 41-60 years age group (Table 1).

**Table 1:** Demographic features of patients presenting with UGIB (N = 730). UGIB: Upper gastrointestinal bleeding.

Characteristics	N (%)
Age (years)	
Mean	49.38
Standard Deviation	14.86
Range	14-99
Gender	
Male	464 (63.6)
Female	266 (36.4)
Age Groups (years)	
<40	206 (28.2)
41-60	387 (53.0)
> 60	137 (18.8)

Table 2 shows the endoscopic findings in patients of UGIB in our study. The most common endoscopic finding was esophageal varices which occurred in more than half of the patients who had UGIB (n = 371). The second most common endoscopic finding after esophageal varices was portal hypertensive gastropathy which was seen in 114 (15.6%) patients. This was followed, in decreasing order of frequency by gastritis (68, 9.3%), cardio-fundal varices (58, 7.9%), duodenal ulcer (26, 3.6%) and other rarer causes (Table 2). While comparing the distribution of endoscopic findings with respect to gender, esophageal varices, gastritis, duodenal ulcers and gastric carcinomas were more likely to be found in male UGIB patients as compared to female patients (p = 0.039). Gastropathy, esophageal ulcer and gastric ulcer were more likely to be found in female UGIB patients.

**Table 2:** Distribution of endoscopic findings in UGIB patients and its frequency with gender (N = 730, male = 464 and female = 266). *p = 0.039 comparing endoscopic findings between male and female patients. **percentages within gender. UGIB: Upper gastrointestinal bleeding.

Endoscopic findings	Gender^*^		Total (%)
	Male (%)^**^	Female (%)^**^	
Esophageal varices	245 (52.8)	126 (47.4)	371 (50.8)
Gastropathy	60 (12.9)	54 (20.3)	114 (15.6)
Gastritis	47 (10.1)	21 (7.9)	68 (9.3)
Cardio-fundal varices	37 (8.0)	21 (7.9)	58 (7.9)
Duodenal ulcer	20 (4.3)	6 (2.3)	26 (3.6)
Esophageal ulcer	9 (1.9)	9 (3.4)	18 (2.5)
Gastric ulcer	8 (1.7)	10 (3.8)	18 (2.5)
Gastric erosions	8 (1.7)	6 (2.3)	14 (1.9)
Duodenitis	8 (1.7)	5 (1.9)	13 (1.8)
Gastric tumour	11 (2.4)	1 (0.4)	12 (1.6)
Esophageal erosions	5 (1.1)	5 (1.9)	10 (1.4)
Esophagitis	3 (0.6)	0 (0)	3 (0.4)
Dieulafoy's lesion	1 (0.2)	2 (0.8)	3 (0.4)
Esophageal tumour	2 (0.4)	0 (0)	2 (0.3)

Table 3 depicts the distribution of endoscopic findings with respect to age of patients. Esophageal varices and cardio-fundal varices were more likely to be found in UGIB patients from middle age group (p = 0.000). While gastritis, duodenal ulcer, gastric erosions and duodenitis were more likely to be found in older (>60 years) UGIB patients (p = 0.000).

**Table 3:** Causes of upper gastrointestinal bleeding in different age groups (N = 730). *p = 0.000 comparing endoscopic findings among different age groups. **percentages within age groups of patients.

Endoscopic findings	Age of the patients^*^			Total N (%)
	<40 years n (%)^**^	41-60 years n (%)^**^	>60 years n (%)^**^	
Esophageal varices	92 (44.7)	220 (56.8)	59 (43.1)	371 (50.8)
Gastropathy	28 (13.6)	63 (16.3)	23 (16.8)	114 (15.6)
Gastritis	33 (16.0)	17 (4.4)	18 (13.1)	68 (9.3)
Cardio-fundal varices	14 (6.8)	38 (9.8)	6 (4.4)	58 (7.9)
Duodenal ulcer	9 (4.4)	9 (2.3)	8 (5.8)	26 (3.6)
Esophageal ulcer	8 (3.9)	9 (2.3)	1 (0.7)	18 (2.5)
Gastric ulcer	7 (3.4)	7 (1.8)	4 (2.9)	18 (2.5)
Gastric erosions	5 (2.4)	5 (1.3)	4 (2.9)	14 (1.9)
Duodenitis	2 (1.0)	5 (1.3)	6 (4.4)	13 (1.8)
Gastric tumour	3 (1.5)	3 (0.8)	6 (4.4)	12 (1.6)
Esophageal erosions	3 (1.5)	6 (1.6)	1 (0.7)	10 (1.4)
Esophagitis	1 (0.5)	1 (0.3)	1 (0.7)	3 (0.4)
Dieulafoy's lesion	0 (0.0)	3 (0.8)	0 (0.0)	3 (0.4)
Esophageal tumour	1 (0.5)	1 (0.3)	0 (0.0)	2 (0.3)

## Discussion

In our study, patient presenting with UGIB had a mean age of 49.38 years. This was slightly lower as compared to mean age of UGIB patients reported in western studies [7,8]. This may depict higher ratio of older population in western world. In our study, UGIB was more common in male patients as compared to females, with a male to female ratio of 1.7:1. This was comparable to the data reported in western studies in which male patient ratio was greater than females [2,9].

The most common and important etiology of UGIB found in our study was bleeding from esophageal varices (50.8%). Other causes of UGIB included portal hypertensive gastropathy, gastritis and gastric erosions. Peptic ulcer is more common than esophageal varices in western world. However, the endoscopic findings of peptic ulcer (gastric and duodenal ulcer) were only found in 6.1% of patients in our study. Similar results were reported in local and international studies as well [10-12]. In contrast to our study findings, several other studies showed peptic ulcer disease as the most common cause of UGIB [13-16].

High prevalence of esophageal varices in our population may be due to the high prevalence of viral hepatitis (Hepatitis B and C) related cirrhosis. Comparable results to our study are also found in other local studies as well. In a local study, 53% of cases had esophageal varices and 20% had peptic ulcer disease [17]. In our study, portal hypertensive gastropathy was the second most common cause of UGIB while in another local study, 64% of cases had esophageal varices and gastric erosions were the second most common cause, i.e., erosive gastritis 15% and PUD 10% of cases [18].

There were some limitations in our study. As our study was carried out at a tertiary care centre, it may not be representative of the general population. Many patients with milder symptoms may not report to the hospital. Also, this was a single center study. Another important thing is that as our hospital is a government run setup, patients mostly belonging to low socio-economic status come here. Multicenter studies targeting clinics providing EGD services should also be taken into account in future studies to further improve our understanding of different etiologies of UGIB.

## Conclusions

It is very essential to have a basic understanding of different conditions causing UGIB. Our study showed that male patients were more likely to present to emergency department with UGIB. Bleeding from esophageal varices was the most important cause of UGIB and bleeding from duodenal ulcer was quite uncommon as compared to the western studies. Esophageal varices, gastritis, duodenal ulcers and gastric carcinomas were more likely to be found in male UGIB patients. Conversely, female UGIB patients were more likely to have findings of gastropathy, esophageal ulcer and gastric ulcer on EGD. Variceal bleeding was more likely to be found in middle age patients. While gastritis, duodenal ulcer, gastric erosions and duodenitis were more likely to be found in older patients.
